# The interrelationships between developmental domains in 3- to 6-year-olds with fine and gross motor developmental risks—results of a prospective dynamic cohort study

**DOI:** 10.1186/s12887-026-06616-w

**Published:** 2026-02-24

**Authors:** Annemarie Köppen, Marco Franze, Wolfgang Hoffmann

**Affiliations:** Institute for Community Medicine, Section Epidemiology of Health Care and Community Health, University Medicine Greifswald, Ellernholzstr. 1-2, Greifswald, 17487 Germany

**Keywords:** Developmental risk, Developmental screening, Preschool, Child, Motor skills, Cognition

## Abstract

**Background:**

Motor skills of school-age children have an impact on learning, school results, and leading a healthy life. However, prevalence rates of motor developmental delays are increasing. Still there is a lack of understanding interrelationships between developmental domains of preschool aged children.

**Methods:**

We used data from the project “GIF MV”, a prospective dynamic cohort study. In this study, cross sectional data was used from the survey year 2023. To detect developmental delays of pre-schoolers a standardized, objective and valid screening method was used (“Dortmund Developmental Screening for Preschools-Revision (DESK 3–6 R)”). We determined associated variables for fine and gross motor developmental risks in preschoolers using Generalized Linear Mixed Models (*n* = 7,542 children).

**Results:**

Fine motor risks were observed in 16.8% of the children (gross motor risks: 12.2%). A gross motor developmental risk was strongest associated with a fine motor developmental risk (and vice versa). Nevertheless, gender and almost all other developmental domains are statistically significantly associated with motor developmental risks, e.g., with developmental risks in the DESK domains “Basic competencies in mathematics” (OR: 4.34, 95% CI: 2.03; 9.27) and “Attention and concentration” (OR: 3.77, 95% CI: 1.8; 7.91).

**Conclusion:**

Since analysis on variables revealed statistically significant associations between motor risks and risks in the remaining developmental domains and gender, activities designed to promote motor skills should include more than just motor developmental domains.

**Trial registration:**

The study was retrospectively registered on 29 October 2018 in the German Clinical Trial Register (ID: DRKS00015134).

**Supplementary Information:**

The online version contains supplementary material available at 10.1186/s12887-026-06616-w.

## Introduction

### Background

Motor skills have an impact on learning [24], cognitive function [20], school results [31], and leading a healthy lifestyle [38]. Especially fine motor skills are necessary to be able to learn how to write [9]. On the other hand, school-age children affected by delayed motor development show high levels of sedentary behaviour [38] and are likely to spend less time engaging in physical activity. Both are risk factors for developing obesity [11, 23], cardiac diseases, and metabolic syndrome [2, 8].

When examining the relation between motor skills and cognition, research results indicate that motor development is associated with cognitive development especially in children with intellectual and developmental disabilities. Among young children not only the physical activity but also the early infant variability of motor skills or the “size of motor repertoire” are associated with IQ at 4 years of age [22]. An Asian study revealed gross motor changes as the second strongest predictor of lower intelligence quotient (IQ) scores in children 2–5 years old (*N* = 423) [10]. Analysing how fine and gross motor skills are influenced by certain factors, sex is also a significant variable (with female sex being a protection factor) [23, 33, 46]. In addition to the sex-specific risks there are also age-group differences: children aged 4.26 to 5.49 years are more affected by delays in motor skills than children aged 6 to 8 years [42]. Hilpert et al. examined the sociocultural influence and identified a lower educational level and migration background (emigration of the child itself or of one or both parents) as risk factors for developing poorer motor skills [1, 23, 37].

In contrast, up to now, the impact of different developmental domains (e.g. cognitive and social-emotional skills) on the motor development of children is unclear. On the one side, Viegas et al. showed that low global cognitive function and little regular physical activity may be independent predictors of delayed gross motor skills, on the other side this study was only carried out on a small sample (*N* = 166) of children in Brazil [47]. Therefore, the small number of cases is associated with large confidence intervals and an imprecise estimate and the transferability of these results for the European region is unclear. Another study revealed that inattention is a predicting variable for a negative change in longitudinally assessed motor skills and that a positive change is predicted by better language skills, cognitive stimulation at home, and a higher family income [35]. However, this study was restricted to children affected by Attention Deficit Hyperactivity Disorder. Therefore, there is a lack of data on the interrelationship of different developmental domains in healthy children affected by motor developmental risks.

This leads to our research question of whether children affected by motor risks simultaneously show low skills in other developmental domains such as language or cognition. Answering this research question is important because it provides evidence how these children can be promoted to catch up with their peers and how a delayed motor development eventually can be prevented.

In doing so, it is necessary to detect motor developmental risks as early and comprehensively as possible in order to be able to start programmes for children at risk well before the school entry age. Preschools in Germany provide an excellent setting for screening because of the high utilization rate among children aged 3 to 5 years (Germany: 91.3%, Mecklenburg Western Pomerania: 94.7%) [41]. Yet, there is a need for a valid, setting-sensitive screening method.

Our study addresses the research gap in the analysis of risk factors during the preschool period. We present results on the impact of gender, cognition, language and social-emotional skills on motor risks based on data from a valid developmental screening and a relatively large sample.

## Methods

### Study design

We used data from the project “GIF MV”, a prospective dynamic cohort study [12, 17]. “GIF M-V” is funded by the Ministry of Education and Daycare Facilities for Children Mecklenburg-Western Pomerania (grant no. 221–157-Br-24). Since 2011 this study has been implemented in more than 240 preschools in the Eastern German federal state of Mecklenburg-Western Pomerania (MWP). Preschools in socially deprived regions are qualified for additional support from the Federal State of MWP. The second requirement for receiving additional funding from the federal state of MWP is the conduction of a standardized developmental screening (DESK 3–6 R) [34], for further details see also [3, 4, 16, 25, 30]. The preschools are selected for the project as follows: the youth welfare offices of each region in MWP determine each preschool’s proportion of children, whose catering costs are covered by social welfare. Inclusion criteria for the project is a proportion above the average.

In this study, cross sectional data is used from the survey year 2023.

### Study region

The federal state MWP is a rural state in northern Germany with a high unemployment rate (MWP: 8%, national average: 6%) [7] and a below average per capital monthly income (MWP: 2,784 €, national average: 4,479 €) [39, 40].

The preschools involved in the study are located in social hotspots in cities (e.g. Rostock, Schwerin, Greifswald, Stralsund) and the rural area in the state.

### Instrument

The DESK 3–6 R is a standardized, reliable, and valid instrument [44] which is highly accepted by teachers in preschools in MWP [15]. The screening assesses age-specific competencies of 3- to 6-year-olds in the domains fine and gross motor skills, social behaviour, cognition, language and communication (different versions for 3-year, 4-year and 5–6-year-old children).

The domain “fine motor” is defined by hand-eye-coordination (see additional file 1, Table S1-Table S3). The domain “gross motor” includes movement coordination and balance (see additional file, Table S1-Table S3). The domain “language and communication” includes prosodic (sound structure), pragmatic (language usage) and linguistic (morphology, sentence structure) competencies. The preschool teacher analyses for example if the child is able to hold a conversation (task in 3- and 4-year olds). The domain “cognition” encompasses logical thinking, retention, understanding and attention (for tasks see Table S4 in the additional file). As an example, the teacher checks if a child recognizes connections in a picture book and describes them. That task is asked for 3- and 4-year-old children. In the domain “basic competencies in written language” the phonological information processing is examined. For example, the child has to recognize words broken down into syllables. For the domain “basic competencies in mathematics” knowledge about quantities and numerical skills are assessed. In this domain the teacher analyses if the child is able to sort quantities by size, for example. The domain “social behaviour” can be broken down into emotional competencies and social competencies [44]. Both competencies are checked for social appropriateness on the group level. One task observed by the preschool teacher is whether or not a child approaches other children on its own initiative. The domains “basic competencies written language”, “basic competencies in mathematics” and “attention and concentration” correspond to the requirements for school entry which are assessed in the DESK version for 5- and 6-year-old children.

The DESK 3–6 R is carried out during 1. a standardized play situation (group tasks performed within the so-called “circus play”), 2. standardized individual tasks (performed with a single child), and 3. observation tasks (rated by the preschool teacher) [44]. The active play takes place in small groups with up to 5 children while the observation tasks are part of the daily routine in the respective child’s preschool group. The implementation of the tasks is clearly described for the teachers in order to ensure a high level of standardization (See Table 1).

**Table 1:** Description of the DESK 3–6 R tasks for concrete preschool teacher rating

Type of task	Task (Example)	Description for the Preschool Teacher
Group play task	Stands in a one-legged stance for at least 5 s in a stable balance (gross motor- 4-year-old children)	The task is completed when the child can maintain balance on both the right and left legs for at least 5 s. If they can only do this on one leg or for less than 5 s, mark “incomplete/inconclusive”. The suspended foot must not be supported by the body, and the knee should be bent at approximately 90 degrees. The child must not hop forward, put their foot down, or hold on to anything during this exercise
Individual task	Knows at least 3 colors (cognition/speech-3-year-old children)	The child can either correctly name at least 3 colors from red, green, blue, yellow, white, black (e.g. “What color is this building block?”) or point to them when asked (e.g. “Show me the red building block”) Solved: The child correctly names or shows 3 colors
Observation task	Paints within given lines (fine motor- 5- and 6-year-old children)	The child paints shapes in a coloring book, for example. The pencil is guided in such a controlled manner that the boundaries are largely adhered to and the shape is largely filled in. It must be clear that the child stops the movement of the pencil before the boundary and traces the shape with the pencil, e.g. following the curves

The proportions of the different task types in each age group are as follows: 3-year-old children: 33.3% circus play, 10.3% individual, 56.4% observation; 4-year-old children: 28.3% circus play, 21.7% individual, 50% observation; 5–6-year-old children: 30.3% circus play, 18% individual, 51.5% observation.

Performance is rated using a 3-step response scale (“yes”, “incomplete/inconclusive”, “no”; tasks assessing social behaviour: “often”, “sometimes”, “rarely/never”).

Preschool authorities and the management staff of DESK-preschools were informed about the project via emails, visits and a central introductory event. Parents were informed with letters and in meetings with the teachers. Before the preschool teachers were able to conduct DESK 3–6 R assessments, they attended a training course explaining and practicing the application of the DESK screening tasks.

### Methods to reduce possible bias

Approx. 50% of the DESK 3–6 R tasks are not merely a teacher´s rating, which is important in order to avoid observer bias. Additionally, all preschool teachers take part in a training course before the DESK 3–6 R tasks are first implemented with the aim of facilitating a standardized implementation. Data on objectivity, reliability and validity are reported [44].

Among other things, Tröster et al. calculated the percentage of concurring judgements (PC): in the version for 3-year-olds, there is very good (PC ≥ 90) or good (PC 80–90) interrater agreement for 77% of the items on both motor skills scales (See Table 2). In contrast, this is only the case for 58% of the items in the cognition/speech domain and 11% of the items in the social behaviour domain. In the version for 4-year-olds, almost all developmental domains have a PC of at least 80 for 80%—88.8% of their development tasks (the social behaviour is an exception: PC of at least 80 for only 22% of the development tasks in this domain). The interrater agreement is even better in the version for 5–6-year-olds. In 5 of 8 developmental domains, a PC of at least 80 is present in 100% of the development tasks. For the remaining domains, this is the case for 62.5% to 75% of the respective development tasks.

**Table 2:** Indices of reliability and objectivity for the fine and gross motor scales

Indices	Fine motor	Gross motor
Interrater agreement	3-year-olds: 97.4	3-year-olds: 92.3
	4-year-olds: 79.4	4-year-olds: 94.1
	5–6-year-olds: 89.1	5–6-year-olds: 83.6
Cronbach’s Alpha	3-year-olds: 0.73	3-year-olds: 0.72
	4-year-olds: 0.71	4-year-olds: 0.73
	5–6-year-olds: 0.75	5–6-year-olds: 0.69

The internal consistency (Cronbach’s Alpha) ranged between 0.69 and 0.92 (See Table 2).

The DESK results correlate strongly with the results of the school entry examination. 84% of children with limited learning preconditions had conspicuous DESK 3–6 R results at 6 years of age. Furthermore, a longitudinal analysis shows that reading (58%), mathematics (70%) and behavioural problems (80%) in second grade were predicted by preschool screening in the year prior to school entry [43, 44].

### Study sample

In the DESK survey year 2023, the developmental screening was conducted for *N* = 7,820 preschool-children. Referring to the DESK domains of fine motor skills and gross motor skills a data set with *N* = 7,542 children was available for analysis. The sample consists of *N* = 3,779 male and *N* = 3,763 female children. The sample comprised *N* = 1,768 3-year-old children, *N* = 2,141 4-year-old children, *N* = 2,388 5-year-old children and *N* = 1,245 6-year-old children.

### Statistical methods

The main results of the screening are stanine-scores based on the number of tasks solved in each DESK domain. A stanine value of 1 represents reasonable grounds to suspect a developmental risk and should trigger a medical examination. A stanine value of 2 is an inconclusive screening result indicating that further observation of the child should occur with a repetition of the DESK 3–6 R after some time has passed. Stanine values between 3 and 9 indicate no developmental risk [44].

The calculation of stanine-scores was performed by the Institute for Community Medicine of University Medicine Greifswald, Germany. DESK stanine scores were dichotomized to divide children at risk from children without developmental risks due to their stanine values (coding: 1 = children at risk, i.e. stanine values 1 and 2 vs. 0 = children without risks, i.e. stanine values 3 to 9).

Prevalence rates of developmental risks in the DESK domains fine and gross motor were calculated for all children (*N* = 7,542) and stratified by sex and age group. Sex differences in prevalence rates were evaluated by means of Pearson’s chi-squared test.

Next, we fitted multilevel linear models (Generalized Linear Mixed Models; GLMM) based on a two-level hierarchy with children's DESK-scores nested within each individual “DESK-preschool” as the contextual-level predictor (random effect covariance type: variance component, i.e. assumption of independence of all random effects; [14, 44, 49]). We calculated GLMM with the dichotomized fine motor and gross motor DESK group status as the outcome variable and the remaining DESK domains as the predictor variables (coding: 1 = children at risk vs. 0 = children without developmental risks). Since we assumed there would be a variation between the “DESK-preschools” in terms of the association between developmental motor risks and risks in the remaining DESK domains, we also assumed that the intercepts vary around the overall model (random intercept model). For the calculation of the GLMM, there was evidence of statistically significant variability in the random effect [21], i.e. preschool affiliation (see Table S10 in the Additional file 1). There was hardly any variation in the random effect for the prediction of gross motor developmental risks among 6-year-olds, so a binary logistic regression (variable selection procedure: enter, i.e. no decision about the order in which variables are entered been made [14]) was calculated in this case.

The DESK stanine scores were calculated using the SAS statistical software package (Version 9, SAS Institute Inc., Cary, USA). IBM SPSS Statistics was used to compute descriptive statistics and multilevel linear models (Version 29, IBM, Armonk, USA). All inference statistics are two-sided with an α error probability of 0.05.

All methods were carried out in accordance with the relevant guidelines and regulations.

## Results

### Prevalence rates

In the DESK domain fine motor skills 16.8% of the children are at risk and in the DESK domain gross motor skills this proportion is 12.2%. While 23.6% of male children are affected by developmental risks in fine motor skills, this is only the case in 10% of female children. With respect to gross motor skills, 14.6% of the boys are affected by developmental risks (girls: 9.8%).

Across sex and age groups, the prevalence rate of developmental risks is highest among 4-year-old boys (fine motor: 33.5%, gross motor: 20.5%) (see Table 3). The prevalence rates increase until 4 years of age and then decline with the lowest rate at 6 years of age. In girls, gross motor developmental risks occur with almost the same prevalence until the age of 5, with lower prevalence among 6-year-olds.

**Table 3:** Prevalence rates of fine and gross motor developmental risks by sex and age group (*N* = 7,542)

Age group, Sex	Fine motor risks	Gross motor risks
	% (N)	% (N)
3		
Male	21.2 (192)	12.6 (114)
Female	12.6 (109)	10.8 (93)
4		
Male	33.5 (347)	20.5 (212)
Female	12.7 (140)	11.4 (126)
5		
Male	20.5 (248)	15.7 (190)
Female	8 (94)	10.6 (125)
6		
Male	16.5 (104)	5.4 (34)
Female	5.5 (34)	3.9 (24)

A chi-square test of independence was performed to evaluate the relationship between sex and fine motor risks, sex and gross motor risks, respectively. The relationship between these variables was significant (exception: outcome gross motor risks in 3-year-olds)

3-year-olds (Outcome: Fine motor risks): χ2 (*1*, *N* = 1,768) = 22.843, *p* <.001

3-year-olds (Outcome: Gross motor risks): χ2 (*1*, *N* = 1,768) = 1.375, *p* =.241

4-year-olds (Outcome: Fine motor risks): χ2 (*1*, *N* = 2,141) = 131.955, *p* <.001

4-year-olds (Outcome: Gross motor risks): χ2 (*1*, *N* = 2,141) = 33.017, *p* <.001

5-year-olds (Outcome: Fine motor risks): χ2 (*1*, *N* = 2,388) = 76.787, *p* <.001

5-year-olds (Outcome: Gross motor risks): χ2 (*1*, *N* = 2,388) = 13.747, *p* <.001

6-year-olds (Outcome: Fine motor risks): χ2 (*1*, *N* = 1,245) = 38.311, *p* <.001

6-year-olds (Outcome: Gross motor risks): χ2 (*1*, *N* = 1,245) = 1.596, *p* =.206

Most of the sex differences are statistically significant (see Table 3). The sex difference is higher in the domain of fine motor skills. This difference can be seen in all age groups and is most pronounced in the 5-year-old children.

The additional file 1 contains further information on descriptive statistics on DESK stanine values and bivariate correlations of the DESK scores.

### Associated variables for motor developmental risks

In all age groups, gross motor developmental risks are the most reliable associated factor for fine motor developmental risks (and vice versa; see Tables 4, 5 and 6). Quantitatively, this effect is highest in 6-year-old boys affected by fine motor developmental risks.

**Table 4:** Multivariate MLM of fine and gross motor developmental risks in 3-to-5-year-olds

	**Fine motor developmental risks**		**Gross motor developmental risks**	
	**OR**	**95% CI**	**OR**	**95% CI**
3-year-olds (n = 1,768)				
Sex (boys; Ref = girls)	1.92	1.39; 2.65	0.83	0.55; 1.25
Developmental risk in DESK domain “Fine motor”	-	-	6.42	4.52; 9.12
Developmental risk in DESK domain “Gross motor”	6.47	4.56; 9.18	-	-
Developmental risk in DESK domain “Cognition/Speech”	3.27	2.35; 4.56	3.12	2.01; 4.84
Developmental risk in DESK domain “Social behaviour”	1.84	1.29; 2.63	1.81	1.12; 2.94
4-year-olds (n = 2,141)				
Sex (boys; Ref = girls)	3.11	2.41; 4.01	1.12	0.85; 1.48
Developmental risk in DESK domain “Fine motor”	-	-	4.98	3.61; 6.89
Developmental risk in DESK domain “Gross motor”	5.03	3.67; 6.89	-	-
Developmental risk in DESK domain “Cognition”	2.6	1.8; 3.74	2.2	1.49; 3.23
Developmental risk in DESK domain “Speech and communication”	1.33	0.93; 1.9	1.54	1.07; 2.22
Developmental risk in DESK domain “Social behaviour”	2.7	2.05; 3.56	1.82	1.31; 2.54
5-year-olds (n = 2,373)				
Sex (boys; Ref = girls)	3.5	2.52; 4.87	1.14	0.82; 1.58
Developmental risk in DESK domain “Fine motor”	-	-	5.35	3.72; 7.7
Developmental risk in DESK domain “Gross motor”	5.56	3.88; 7.96	-	-
Developmental risk in DESK domain “Speech and communication”	2.02	1.35; 3.02	1.51	1.06; 2.15
Developmental risk in DESK domain “Basic competencies in written language”	2.08	1.43; 3.01	1.85	1.28; 2.68
Developmental risk in DESK domain “Basic competencies in mathematics”	2.36	1.59; 3.5	1.99	1.34; 2.94
Developmental risk in DESK domain “Attention and concentration”	3.07	2.01; 4.68	1.22	0.8; 1.85
Developmental risk in DESK domain “Social competence”	1.02	0.67; 1.55	1.45	1; 2.1
Developmental risk in DESK domain “Social interaction”	1.75	1.09; 2.83	1.06	0.69; 1.63

Coding of predictor and outcome variable: 1 = children at risk, 0 = children without developmental risks

**Table 5:** Multivariate MLM of fine motor developmental risks in 6-year-olds

*6-year-olds (n* = *1.245)*	OR	95% CI
Sex (boys; Ref = girls)	**3.48**	2.11; 5.74
Developmental risk in DESK domain “Fine motor”	-	-
Developmental risk in DESK domain “Gross motor”	**6.87**	3.79; 12.46
Developmental risk in DESK domain “Speech and communication”	**2.17**	1.22; 3.85
Developmental risk in DESK domain “Basic competencies in written language”	**2.6**	1.53; 4.41
Developmental risk in DESK domain “Basic competencies in mathematics”	**2.3**	1.38; 3.83
Developmental risk in DESK domain “Attention and concentration”	**2.57**	1.43; 4.63
Developmental risk in DESK domain “Social competence”	1.08	0.59; 1.98
Developmental risk in DESK domain “Social interaction”	1.42	0.89; 2.27

Coding of predictor and outcome variable: 1 = children at risk, 0 = children without developmental risks

**Table 6:** Binary logistic regression of gross motor developmental risks in 6-year-olds

*6-year-olds (n* = *1,245)*	OR	95% CI
Sex (boys; Ref = girls)	0.67	0.35; 1.3
Developmental risk in DESK domain “Fine motor”	**6.81**	3.29; 14.06
Developmental risk in DESK domain “Gross motor”	**-**	**-**
Developmental risk in DESK domain “Speech and communication”	0.81	0.33; 1.98
Developmental risk in DESK domain “Basic competencies in written language”	0.8	0.34; 1.98
Developmental risk in DESK domain “Basic competencies in mathematics”	**4.34**	2.03; 9.27
Developmental risk in DESK domain “Attention and concentration”	**3.77**	1.8; 7.91
Developmental risk in DESK domain “Social competence”	1	0.44; 2.24
Developmental risk in DESK domain “Social interaction”	**2.34**	1.18; 4.64

Coding of predictor and outcome variable: 1 = children at risk, 0 = children without developmental risks

The second strongest associated factor of fine or gross motor developmental risks in 3-year-old children can be seen in cognition/speech. In 4-year-olds, in particular risks in social behaviour and cognition also seem to be important associated factors for a fine or gross motor developmental risk. In 5-/6-year-olds, the second strongest association is observed with the domains attention and concentration (5- years olds) or basic competencies in written language (6-years olds) for fine motor risks and mathematics, respectively for gross motor risks (5- and 6-year olds).

Throughout all age groups a strong association can be seen in male gender as a risk factor especially in the age group of 5–6-year-old children.

Depending on the model, the ICC (intraclass coefficient) ranges between 0.047 and 0.08. Thus, between 4.7% and 8% of the variance in fine and gross motor developmental risks can be explained by differences between preschools.

## Discussion

This study reveals that the remaining developmental domains cognition, language and social skills all are associated with motor development in preschool-age children.

As expected, the highest associations were observed with low gross motor skills (outcome: fine motor developmental risks), and low fine motor skills (outcome: gross motor developmental risks).

Among the 3-year-old children the association of cognition/speech or cognition on both fine and gross developmental risks is higher than among the 4-year-old children. A study of brain development during early life and the preschool years [6] provides an explanation for this effect. Even though many brain patterning processes are completed at the time of birth, other biological developments take place in the preschool years. While the prenatal period is marked by the production and migration of neurons, the proliferation and migration of glia cells and differentiation of astrocytes are postnatal processes. These processes are critical for the functional maturation of developing neural circuits. In younger children the within-network correlation is higher than the across-network correlation between the two neuronal networks ToM (“Theory of Mind”-network, e.g. reasoning about others’ minds) and pain [36]. For the two networks a rising positive within-network correlation and a rising negative across-network correlation can be observed until adulthood is reached. To measure the activation in children’s brains external stimulation was used, e.g. watching a movie which activates both brain regions in adults. These two networks seem to already be independent of each other by the time children are 3. In older children and adults ToM and the activation of pain networks are negatively correlated. A similar relation may be present for motor and cognition brain networks, which can be seen in the declining trend of the association from age 3 to age 6. In contradiction to this, the review of Haapala showed that there was an association between motor skills and cognition even in school-age children, although the effect was mainly based on higher cardiorespiratory fitness [20]. Cross-sectional studies suggest a reason for this could be more efficient cognitive processing at the neuroelectric level but to evaluate causal pathways there is a need for longitudinal studies.

For the 5- and 6-year-old children the school requirements play a dominant role in influencing motor developmental risks. Low basic competencies in mathematics and written language are associated with both fine and gross motor developmental risks. This can explain the association of motor development and school results especially in mathematics and reading and writing skills. As expected, the general learning requirements, namely attention/concentration [44] seem to be important associated factors of motor developmental risks, thus confirming the results observed for children with Attention Deficit Hyperactivity Disorder. Peyre et al. suggested there is a relation between inattention and a negative change in motor skills [35]. However, Peyre et al. used a parental questionnaire which probably yields less valid results than the standardized DESK 3–6 R screening. Risks related to social skills are less associated for motor developmental risks and the prediction decreases as children get older. This can be explained by the fact that the cognition processes for social skills and motor skills are rather independent for primary school-age children [26].

Our study also reveals, that the influence of sex on fine motor risks seems to be more important than age differences. A possible explanation can be found in the different sex-specific development [13]. In our study the sex difference is rising from 3 to 5 years of age, especially in fine motor risks, and is highest in 5-year-old children. The Review of Etchell et al. summarizes the research as inconsistent in evidence for sex differences in brain structure and function, further research is needed.

Motor developmental delays in preschool-age children are increasing in prevalence [27]. The prevalence rates of fine motor developmental risks (16.8%) and gross motor developmental risks (12.2%) in our analysis are consistent with the rates observed by Stich et al. (14.4%) in Bavarian preschoolers in 2009 (*N* = 13,876) [42]. In the MOMO study conducted by the Robert Koch Institute [48] prevalence rates seem to have remained stable in Germany during the most recent survey waves (survey wave 2: 2014–2019). International data suggests different prevalence rates of gross motor delay (overall between 3–11%) [46] with highest prevalence rate of 77% in the United States [5]. Fine motor risks were reported with a prevalence rate of 14–17% in Austria [19], 16.5% in Palestine [1] and 4.3% in Iran [37]. Even though we used data from after the first COVID-19 wave in Germany, the rates seem to be comparable with rates before the pandemic in Europe. The most affected children were born only one year before the first COVID-19 wave, so their data before the age of 3 years are not recorded in the DESK 3–6 R and we are therefore not able to compare the skills before and after COVID-19. In order to obtain an accurate picture, future studies should use an instrument that can also be applied to younger children. Nevertheless, the DESK screening results show that at least every eighth and in some cases as many as every sixth child is affected by a motor developmental risk and needs special attention and promotion to catch up with the peer group. Especially male children aged 4 or 5 years are at risk of developing motor developmental delays. This finding confirms former studies [42] and adds further evidence underlining the importance of this particular age group.

As a consequence, a sex-sensitive and age-specific intervention concept is needed to prevent motor developmental risks. This intervention can be implemented in preschools as the utilisation rate is particularly high among children between the ages of 3 and 6 years old [41]. In preschools, children are able to observe their peers in everyday life and learn new skills directly by copying other children. Moreover, the preschool teachers’ promoting activities can be integrated into the preschool routine so that children can easily adopt them and then use the skills they have trained to continue practising at home. This could reduce the time required for promoting activities addressing children with developmental risks and prevention could be delivered at the group level during the usual preschool routines. This is of great importance as there is an increased focus on efficiency with preschools facing a shortage of skilled preschool teachers.

### Limitations and strengths

The limitations of our study are as follows:

1. The results cannot be fully generalised.

Table S10 in the additional file shows that there is a statistically significant variation over different preschools in the random effect. On the one hand, it is therefore important to consider the context variable of preschool affiliation. This is plausible in view of the fact that preschools differ from one another in terms of their educational quality [45]. On the other hand, the results for the ICC show that only a maximum of 8% of the variance in the outcome can be explained by differences in preschools alone. This result supports the fact that the study involves preschools with a common specific characteristic (i.e. a difficult socio-spatial location). Thus, the results cannot be readily transferred to all preschools in MWP and the reported prevalence rates of developmental risks may constitute an overrepresentation when compared to other regions in MWP. For this reason, a study involving institutions in better socio-spatial locations would be desirable in order to determine the replicability of the results.

(2) There is a lack of data of cost coverage by social welfare in every age group. That leads to possible differences in the proportion of socially disadvantaged children in every age group. Following the different prevalence rates of motor developmental risks can be influenced by that effect. Further studies should collect such data.
(3) Another limitation concerns the lack of data on children aged 0 to 2 years.

The strengths of our study are as follows:

1. Since our study is based on a standardized assessment of children´s competencies conducted by preschool teachers who have received prior training it has a higher objectivity than studies which used parental questionnaires as the only source of information [32].
2. Our results are important because of the rather large data base of children in the DESK screening. This favours comparatively narrow confidence intervals, thus leading to a more precise estimate.
3. Previous published results suggest risk factors but there are limitations associated with the sample size and/or the study focus, e.g. cognitive function, language development, and attention [22].
4. The screening is not only focused on motor skills but also includes the assessment of other developmental domains including cognition, language and speech, and social skills. Several influencing factors for motor development screened by other studies depend on the degree of support and opportunities offered to the children. Most of the previous studies do not reflect a potential influence of other domains on motor competencies. Instead, these studies concentrate on recommendations to increase children’s *physical activity*, e.g. [18, 23].
5. Furthermore, our study is unique in Germany as we have a high preschool utilization rate, with annually 7,000—8,000 children from approx. 160 preschools involved and, in addition, the families have comparable socioeconomic backgrounds.
6. Individual support helps the children to catch up to the peer group so that sociocultural differences can be alleviated. The need for our screening is clear especially with such a social focus (see for example [28, 29]) and the follow-up studies will hopefully show a declining trend in terms of developmental risks due to the individual support provided to the affected children.

## Conclusion

Supporting preschoolers in their motor development should simultaneously take into consideration all developmental domains and be sex-sensitive. For children with motor developmental risks preschool teachers should not limit their focus to the advancement of the respective motor skills. Rather, they should include activities to promote cognition and social skills to foster the simultaneous development of these deeply interconnected domains. Thus, our results support calls for ‘networked promotion’, which aims to address more than one area of competence, e.g. the promotion of language skills through movement [49].

Children with developmental risks in different domains could be trained together. This could make it easier to support and promote children within the daily routine of preschools, which is especially relevant in regions with a shortage of skilled preschool teachers.

## Supplementary Information

Supplementary Material 1.

## Data Availability

The datasets generated and analyzed during the current study are not publicly available after consultation with the data protection commissioner of Mecklenburg-Western Pomerania due to data protection reasons and contractual regulations. For further information, please contact Dr. Marco Franze (Email: marco.franze@uni-greifswald.de).

## Abbreviations

DESK 3–6 R: Dortmund Developmental Screening for Preschools-Revision
MWP: Mecklenburg Western Pomerania
PC: Concurring judgements
GLMM: Generalized Linear Mixed Models
ICC: Intraclass coefficient
