# Supplementary material for: The interrelationships between developmental domains in 3- to 6-year-olds with fine and gross motor developmental risks—results of a prospective dynamic cohort study
Source: BMC Pediatr. 2026 Feb 24;26:255. doi: 10.1186/s12887-026-06616-w (PMC13045053; doi:10.1186/s12887-026-06616-w)
Supplement: Supplementary file 1 — Supplementary Material 1. [file 12887_2026_6616_MOESM1_ESM.docx]

**The interrelationships between developmental domains in 3- to 6-year olds with fine and gross motor developmental risks- results of a prospective dynamic cohort study**

**Additional file 1**

- S1-S3 Translated motor tasks of DESK 3-6 R
- S4 Translated cognition tasks of DESK 3-6 R
- S5 Descriptive statistics on DESK stanine values
- S6-S9 Bivariate correlations (Spearman´s rho) of the DESK scores
- S10 Variability of the random effect in the unconditional models (no predictor variables)

**Table S1: Translated motor tasks of DESK R for 3-year-old children**

| **Fine motor tasks** | **Gross motor tasks** |
| --- | --- |
| Draws a cross | Hops on one leg |
| Traces a pre-drawn cross with the pencil | Performs 5 consecutive final jumps in a row |
| Folds a sheet of paper in the middle | Jumps a distance of at least 20cm in a final jump |
| Pours a half-full cup of water into another cup and back | Stands in a one-legged stance in safe balance for at least 3 seconds |
| Shapes a snake out of a ball of dough in the size of a walnut | Catches a thrown ball |
| Opens a box of matches | Jumps over a height of 5 cm in alterning steps without falling down |
| Can handle the scissors | Walks at least 3 steps down or up a flight of stairs at an adult pace |
| Opens or closes buttons | Rides a tricycle, balance bike or scooter |
| Tears pieces of paper | Balances on a beam |

**Table S2: Translated motor tasks of DESK R for 4-year-old children**

| **Fine motor tasks** | **Gross motor tasks** |
| --- | --- |
| Draws a cross | Performs 5 consecutive final jumps in a row |
| Traces a pre-drawn cross with the pencil | Hops on one leg |
| Touches the thumb alternately with the fingers of one hand | Jumps three times in a row into the side straddle position and back |
| Winds up a ball of wool | Balances forward in a toe-to-toe gait |
| Opens or closes a button | Stands in a one-legged stance in safe balance for at least 5 seconds |
| Cuts a straight line with scissors | Runs backwards |
| Paints within given lines | Jumps back and forth sideways over a rope with both legs at the same time |
| Tears scraps of paper | Performs crossover movements |
| Holds a pen correctly (three-point grip) | Can interrupt movements |

**Table S3: Translated motor tasks of DESK R in 5- and 6-year-old children**

| **Fine motor tasks** | **Gross motor tasks** |
| --- | --- |
| Writes down letters | Balances forward in a toe-to-toe gait |
| Touches the thumb alternately with the fingers of one hand | Balances backwards in a toe-to-toe gait |
| Cuts out a circle with the scissors | Stands in a one-legged stance in safe balance for at least 10 seconds |
| Buttons up a shirt, blouse or jacket | Jumps back and forth sideways over a rope with both legs at the same time |
| Draws within given lines | Performs crossover movements |
| Traces a horizontal figure eight with the pencil | Catches a thrown ball with both hands without stopping it with the upper body |
| Sorts beads into a cup with both hands at the same time | Bounces a ball |
| Draws a star with at least 5 prongs | Can interrupt movements |

**Table S4: Translated cognition tasks of DESK 3-6 R**

| **Type of task** | **3-year old children** | **4-year old children** | **5 and 6-year old children** |
| --- | --- | --- | --- |
| Play situation | Repeats a sentence | - | Recites 4-syllable “magic words” |
|  |  |  | Finds rhyming words |
|  |  |  | Repeats 4-digit number sequences |
|  |  |  | Claps the syllables of 4-syllable words |
|  |  |  | Recognizes words broken down into syllables |
|  |  |  | Can complete words when a sound is missing |
|  |  |  | Recognizes which word is longer out of 3 pairs of words |
|  |  |  | Repeats a whole sentence |
|  |  |  | Reads 4 numbers correctly from the numbers 1-9 |
| Individual task | Answers simple questions about “when”, “what”, and “why” | Classifies animals according to characteristics | Sorts objects according to 2 characteristics |
|  | Knows at least 3 colours | Sorts objects by a characteristic | Names predecessor and successor numbers in the range up to 10 |
|  | Knows the opposites of “small”, “cold”, “bright” and “sour” | Understands and uses quantity terms from 1 to 4 | Arranges objects in a row according to size |
|  | Follows 3 simple prompts in the correct order | Describes the sequence of steps that belong to a sequence of actions | Sorts sets by size |
|  |  | Sorts sets by size | Recognizes that a quantity remains the same even when it is pushed together or apart |
|  |  | Counts the objects in an unordered set up to 10 | Solves simple and illustrative addition and subtraction problems within the number range up to 6 |
| Observation task | Speaks grammatically correct 3-word sentences | Recognizes connections in a picture book and describes them | Can count a set of 5 objects with the eyes without using the fingers to help |
|  | Can name simple activities | Can recite rhymes or sing song lyrics |  |
|  | Uses the plural | Can correctly place a rolled number on the game board |  |
|  | Connects 2 sentences with a junction (e.g. and) |  |  |
|  | Can hold a conversation |  |  |
|  | Recognizes connections in a picture book and describes them |  |  |
|  | Has an inconspicuous pronunciation |  |  |

Note: 3-year-olds: domain-cognition/speech, 4-year-olds: domain cognition, 5-6-year-olds: domains basic competencies in written language and mathematics

**Table S5: Descriptive statistics on DESK stanine values**

|  | **Percentiles** | | |
| --- | --- | --- | --- |
|  | **25** | **50** | **75** |
| **3 year old (n = 1,768)** | | | |
| **Fine motor** | 3 | 5 | 6,5 |
| **Gross motor** | 4 | 5 | 6,5 |
| **Cognition / Speech** | 2 | 4 | 6,5 |
| **Social behaviour** | 3 | 5 | 6,5 |
| **4 year old (n = 2,141)** | | | |
| **Fine motor** | 3 | 5 | 6,5 |
| **Gross motor** | 3 | 5 | 8,5 |
| **Cognition** | 2 | 5 | 6.5 |
| **Speech and communication** | 2 | 5 | 8.5 |
| **Social behavior** | 3 | 5 | 6.5 |
| **5 year old (n = 2,373)** | | | |
| **Fine motor** | 4 | 5 | 6.5 |
| **Gross motor** | 4 | 5 | 8.5 |
| **Speech and communication** | 2 | 4 | 8.5 |
| **Basic competencies in written language** | 3 | 5 | 6.5 |
| **Basic competencies in mathematics** | 3 | 5 | 6.5 |
| **Attention and concentration** | 3 | 5 | 8.5 |
| **Social competence** | 3 | 5 | 8.5 |
| **Social interaction** | 3 | 5 | 8.5 |
| **6 year old (n = 1,245)** | | | |
| **Fine motor** | 4 | 5 | 8.5 |
| **Gross motor** | 5 | 6.5 | 8.5 |
| **Speech and communication** | 2 | 4 | 8.5 |
| **Basic competencies in written language** | 2 | 5 | 8.5 |
| **Basic competencies in mathematics** | 3 | 5 | 8.5 |
| **Attention and concentration** | 4 | 6.5 | 8.5 |
| **Social competence** | 4 | 6.5 | 8.5 |
| **Social interaction** | 3 | 5 | 8.5 |

^Note: In this developmental screening the stanine scores 6-7 and 8-9 are combined into 1 category each. In the data this is represented with the value 6.5, or 8.5, respectively.^

**Table S6: Bivariate correlations (Spearman´s rho) of the DESK scores**

|  | **FMO** | **GMO** | **COG / SP** | **SOCBEHAV** |
| --- | --- | --- | --- | --- |
| **FMO** | 1 |  |  |  |
| **GMO** | 0.48 | 1 |  |  |
| **COG / SP** | 0.45 | 0.42 | 1 |  |
| **SOCBEHAV** | 0.47 | 0.43 | 0.74 | 1 |

^Note: 3-year-olds, n = 1,768. FMO: Fine motor; GMO: Gross Motor; COG / SP: Cognition/Speech; SOCBEHAV: Social behaviour. All correlations significant at the 0.01 level.^

**Table S7: Bivariate correlations (Spearman´s rho) of the DESK scores**

|  | **FMO** | **GMO** | **SP_COMM** | **COG** | **SOCBEHAV** |
| --- | --- | --- | --- | --- | --- |
| **FMO** | 1 |  |  |  |  |
| **GMO** | 0.52 | 1 |  |  |  |
| **SP_COMM** | 0.43 | 0.35 | 1 |  |  |
| **COG** | 0.52 | 0.44 | 0.76 | 1 |  |
| **SOCBEHAV** | 0.44 | 0.33 | 0.47 | 0.5 | 1 |

^Note: 4-year-olds, n = 2,141. FMO: Fine motor; GMO: Gross Motor; SP_COMM: Speech and communication; COG: Cognition; SOCBEHAV: Social behaviour. All correlations significant at the 0.01 level.^

**Table S8: Bivariate correlations (Spearman´s rho) of the DESK scores**

|  | **FMO** | **GMO** | **SP_ COMM** | **BASCOMP_ WRITTEN** | **BASCOMP_ MATHS** | **ATT _CONC** | **SOC _COMP** | **SOC _INTER** |
| --- | --- | --- | --- | --- | --- | --- | --- | --- |
| **FMO** | 1 |  |  |  |  |  |  |  |
| **GMO** | 0.51 | 1 |  |  |  |  |  |  |
| **SP_COMM** | 0.45 | 0.34 | 1 |  |  |  |  |  |
| **BASCOMP_WRITTEN** | 0.52 | 0.41 | 0.68 | 1 |  |  |  |  |
| **BASCOMP_MATHS** | 0.53 | 0.44 | 0.59 | 0.64 | 1 |  |  |  |
| **ATT_CONC** | 0.45 | 0.33 | 0.35 | 0.37 | 0.36 | 1 |  |  |
| **SOC_COMP** | 0.36 | 0.26 | 0.3 | 0.29 | 0.26 | 0.66 | 1 |  |
| **SOC_INTER** | 0.38 | 0.35 | 0.51 | 0.44 | 0.41 | 0.34 | 0.33 | 1 |

^Note: 5-year-olds, n = 2,373. FMO: Fine motor; GMO: Gross Motor; COG / SP: Cognition/Speech; SOCBEHAV: Social behaviour; COG: Cognition; SP_COMM: Speech and communication; BASCOMP_WRITTEN: Basic competencies in written language; BASCOMP_MATHS: Basic competencies in mathematics; ATT_CONC: Attention and concentration; SOC_COMP: Social competence; SOC_INTER: Social interaction. All correlations significant at the 0.01 level.^

**Table S9: Bivariate correlations (Spearman´s rho) of the DESK scores**

|  | **FMO** | **GMO** | **SP_ COMM** | **BASCOMP_ WRITTEN** | **BASCOMP_ MATHS** | **ATT _CONC** | **SOC _COMP** | **SOC _INTER** |
| --- | --- | --- | --- | --- | --- | --- | --- | --- |
| **FMO** | 1 |  |  |  |  |  |  |  |
| **GMO** | 0.45 | 1 |  |  |  |  |  |  |
| **SP_COMM** | 0.4 | 0.31 | 1 |  |  |  |  |  |
| **BASCOMP_WRITTEN** | 0.47 | 0.39 | 0.68 | 1 |  |  |  |  |
| **BASCOMP_MATHS** | 0.45 | 0.4 | 0.56 | 0.63 | 1 |  |  |  |
| **ATT_CONC** | 0.42 | 0.34 | 0.38 | 0.36 | 0.36 | 1 |  |  |
| **SOC_COMP** | 0.33 | 0.26 | 0.3 | 0.28 | 0.28 | 0.68 | 1 |  |
| **SOC_INTER** | 0.37 | 0.31 | 0.51 | 0.44 | 0.41 | 0.34 | 0.34 | 1 |

^Note: 6-year-olds, n = 1,245. FMO: Fine motor; GMO: Gross Motor; COG / SP: Cognition/Speech; SOCBEHAV: Social behaviour; COG: Cognition; SP_COMM: Speech and communication; BASCOMP_WRITTEN: Basic competencies in written language; BASCOMP_MATHS: Basic competencies in mathematics; ATT_CONC: Attention and concentration; SOC_COMP: Social competence; SOC_INTER: Social interaction. All correlations significant at the 0.01 level.^

**Table S10: Variability of the random effect in the unconditional models (no predictor variables)**

|  | **Outcome: Fine motor developmental risks** | | | **Outcome: Gross motor** | | |
| --- | --- | --- | --- | --- | --- | --- |
|  | **Var(Intercept)** | **95% CI** | **p** | **Var(Intercept)** | **95% CI** | **p** |
| **3-year-old** | 0.42 | 0.24, 0.75 | < 0.001 | 0.44 | 0.24, 0.82 | 0.001 |
| **4-year-old** | 0.42 | 0.26, 0.69 | < 0.001 | 0.61 | 0.38, 0.97 | < 0.001 |
| **5-year-old** | 0.41 | 0.25, 0.69 | < 0.001 | 0.67 | 0.44, 1.04 | < 0.001 |
| **6-year-old** | 0.77 | 0.44, 1.37 | < 0.001 | * | | |

* Note: Not reported due to a not positive definite Hessian matrix.
